# Heat Transfer Analysis of Nanofluid Flow in a Rotating System with Magnetic Field Using an Intelligent Strength Stochastic-Driven Approach

**DOI:** 10.3390/nano12132273

**Published:** 2022-07-01

**Authors:** Kamsing Nonlaopon, Naveed Ahmad Khan, Muhammad Sulaiman, Fahad Sameer Alshammari, Ghaylen Laouini

**Affiliations:** 1 Department of Mathematics, Faculty of Science, Khon Kaen University, Khon Kaen 40002, Thailand; nkamsi@kku.ac.th; 2 Department of Mathematics, Abdul Wali Khan University, Mardan 23200, Pakistan; ahmednaveed854477@gmail.com; 3 Department of Mathematics, College of Science and Humanities in Alkharj, Prince Sattam Bin Abdulaziz University, Al-Kharj 11942, Saudi Arabia; 4 College of Engineering and Technology, American University of the Middle East, Egaila 54200, Kuwait; ghaylen.laouini@aum.edu.kw

**Keywords:** two-phase nanofluid flow, heat transfer, magnetic field, horizontal plates, Nusselt number, skin-friction coefficient, artificial intelligence, soft computing

## Abstract

This paper investigates the heat transfer of two-phase nanofluid flow between horizontal plates in a rotating system with a magnetic field and external forces. The basic continuity and momentum equations are considered to formulate the governing mathematical model of the problem. Furthermore, certain similarity transformations are used to reduce a governing system of non-linear partial differential equations (PDEs) into a non-linear system of ordinary differential equations. Moreover, an efficient stochastic technique based on feed-forward neural networks (FFNNs) with a back-propagated Levenberg–Marquardt (BLM) algorithm is developed to examine the effect of variations in various parameters on velocity, gravitational acceleration, temperature, and concentration profiles of the nanofluid. To validate the accuracy, efficiency, and computational complexity of the FFNN–BLM algorithm, different performance functions are defined based on mean absolute deviations (MAD), error in Nash–Sutcliffe efficiency (ENSE), and Theil’s inequality coefficient (TIC). The approximate solutions achieved by the proposed technique are validated by comparing with the least square method (LSM), machine learning algorithms such as NARX-LM, and numerical solutions by the Runge–Kutta–Fehlberg method (RKFM). The results demonstrate that the mean percentage error in our solutions and values of ENSE, TIC, and MAD is almost zero, showing the design algorithm’s robustness and correctness.

## 1. Introduction

Nanofluid is a term, first used by Choi [1] in 1995, which refers to a particular class of heat transfer fluids with some unique thermal properties. It consists of a base fluid and nanoparticles, which are nanometer-sized fragments of substances suspended in a traditional fluid with diameters ranging from 1 to 100 nm. In addition, nanofluids are made up of nanoparticles with a variety of base fluids, including kerosene, polymeric solutions, oil, water, biofluids, ethylene-glycol, and lubricants [2]. The suspended ultrafine particles change the base fluid’s transport properties and heat transfer performance. The study of heat transfer in nanofluids is of great importance in engineering, applied physics, and industrial applications, such as the cooling of microchips, geothermal power extraction, nuclear reactors, distant vehicular nanofluids, nano-drug delivery, smart fluids, and nanofluid detergents [3]. In light of these significant applications, a number of researchers have discussed nanofluids in different geometrical configurations. As part of an exhaustive study of convective transport in a nanofluid, based on MIT research, Buongiorno [4] looked at the seven slip processes that cause the base fluid and nanoparticles to have a relative velocity. Kuznetsov and Nield [5] investigated the effect of nanoparticles on the natural convection flow of the boundary layer through a vertical plate using Brownian motion and thermophoresis. Khan et al. [6] explored the natural convection of a non-Newtonian nanofluid containing gyrotactic microorganisms in a porous medium along with a static plate. Nanofluids improve the heat transfer and also increase the resistance of the flow. In this regard, comprehensive performance indices, including thermal efficiency, exergy efficiency, and entropy generation have been extensively studied by C. Qi [7,8,9,10].

The magnetohydrodynamic (MHD) flow of nanofluids has attracted the interest of various researchers due to its numerous uses in physics, agriculture, medicine, petroleum industries, and engineering. MHD generators, rotating machines, micro-polar fluid flow, electronic storage components, viscometry, lubrications, turbomachines, physical oceanography processes, pumps, and reactor chemical vapor deposition are some examples of MHD fluids [11,12,13]. Nanofluids subjected to a magnetic field essentially modify the heat transfer by maneuvering the suspended nanoparticles [14,15]. Pal and Mondal [16] investigated the influence of viscous-Ohmic dissipations and a magnetic field on the convective-radiative boundary layer flow of nanofluids caused by non-linear stretching/shrinking sheets. They concluded that skin friction and the volume fraction coefficient increase with decrease in the magnetic parameter. Ghadikolaei [17] studied the effect of a magnetic field on the stagnation flow of hybrid TiO_2_-Cu/water nanofluid spanning an expanding surface. Their results indicated that a positive increment in the magnetic field decreased the skin friction coefficient and caused an increase in the Prandtl number. Hosseinzadeh [18] studied the effect of magnetic field and radiation on the hybrid fluid in an octagonal porous medium. Chemically reacting MHD 3D Maxwell nanofluid flow subjected to temperature-dependent transposition was investigated by Ahmad [19]. Rashidi [20] investigated the combined effects of nanoparticles and an irresistible field on a micropolar fluid running between two parallel coaxial porous plates under uniform pumping. They discovered that an increase in these parameters caused an increase in the heat transfer rate at the lower plate.

Generally, the mathematical models governing the two-phase and three-phase flow of nanofluids subjected to magnetic fields are non-linear in nature [21]. Therefore, various numerical and analytical techniques have been developed to solve such models. The finite element method (FEM) was used by Bhargava [22,23] to examine the convection of a mixed micropolar fluid generated by the stretching of a porous sheet. The stagnation point flow over a permeable surface under the influence of magnetohydrodynamics (MHD) was calculated by Bhatti et al. [24], applying a successive linearization technique. The analytical solutions for the boundary layer flow of a nanofluid were examined by Hassani [25], using a homotopy analysis method (HAM). The convection of nanofluid flow in a horizontal layer of finite depth was studied by Wakif et al. [26] using a Wakif–Galerkin weighted residuals method (WGWRM). An Akbari–Ganji method (AGM) [27] was implemented to study a nanofluid’s heat and mass transfer in the presence of a magnetic field. Mosayebidorcheh used the differential transformation method (DTM) to analyze the turbulent flow of an MHD Couette nanofluid [28,29]. S. Gupta [30] investigated the three-dimensional flow of an Oldroyd-B nanofluid over a bidirectional stretching sheet using hybridization of DTM and the Padé approximation. Hatami [31,32] studied nanofluid flow and heat transfer when subjected to a magnetic field and flowing between parallel plates. An MHD nanofluid squeezing flow analysis under the influence of slip boundary conditions was investigated by Sobamowo et al. [33] using a variation of parameter method (VPM). S. Haider [34] used an optimal homotopy analysis method to study the Stefan blowing impact in the presence of Arrhenius activation energy, heat radiation, and chemical reaction of the unsteady MHD nanofluid. Based on this brief overview of the literature, it can be seen that the methods described are purely gradient-based and use a traditional deterministic approach to solve non-linear models. Instead, we have used neural networks that do not require any prior information about the function and its gradient. Neural networks can avoid singularity and learn by themselves and produce output that is not limited to the input provided to them. Therefore, ANNs are considered to be a better alternative for the approximation of differential equations corresponding to real-world phenomena.

Recently, stochastic meta-heuristic and heuristic techniques have been developed to solve a variety of non-linear system problems, such as imbibition phenomena [35,36], bath of wire by Oldroyd 8-constant fluid [37], electrohydrodynamic (EHD) fluid flow analysis with an ion drag configuration in a circular cylindrical conduit [38], the flow of non-Newtonian Johnson–Segalman fluid [39], chaos-based secure wireless communications [40,41], and thermal engineering problems [42,43]. These recent studies on stochastic techniques motivated the authors to incorporate and exploit the strength of artificial neural networks with optimization techniques to study the heat transfer of two-phase nanofluid flow subjected to a magnetic field. Some novel features of this study are listed below:

- This study analyzes the mathematical model of heat transfer of nanofluid flow with a magnetic field by implementing and utilizing the computational strength of feed-forward artificial neural networks with a back-propagated Levenberg–Marquardt (BLM) algorithm.
- A proposed FFNN–BLM technique is exploited to examine the heat transfer, velocity, acceleration, and concentration profiles of a nanofluid by varying the magnetic parameter, Prandtl number, rotation parameter, thermophoresis, and Brownian motion parameter.
- To validate the accuracy and efficiency of the proposed technique, the results are compared with numerical solutions by the Runge–Kutta–Fehlberg method, least square method, and other machine learning algorithms, such as the non-linear autoregressive exogenous neural network model.
- Performance functions, such as MAD, TIC, and ENSE are formulated to study the errors and deviations in the solutions to assess the value of the proposed algorithm.

## 2. Mathematical Formulation

**Figure 1:**
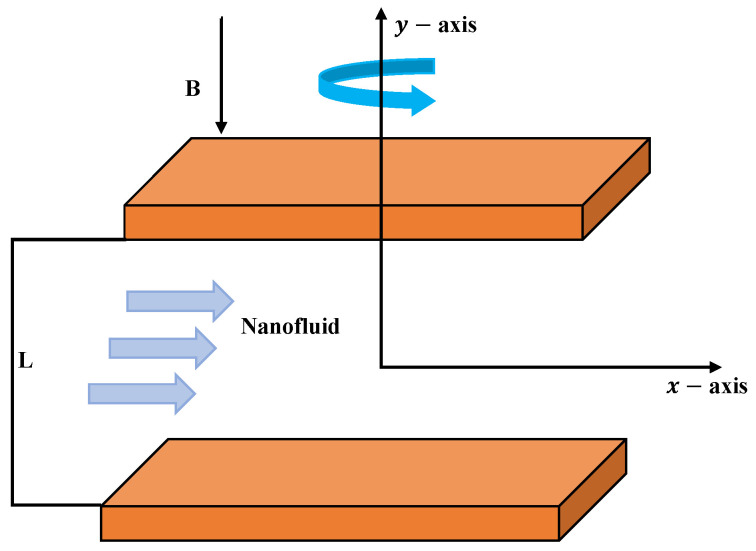
Two-phase flow of a nanofluid in horizontal plates.

We considered the two-dimensional flow of nanofluids rotating between two horizontal plates subjected to a magnetic field as shown in Figure 1. The magnetic field *B* is applied, and the fluid rotates with uniform velocity Ω. The boundary layer equations of continuity, momentum, mass, energy and heat transfer are used to regulate the subjected model, which is expressed as

(1) $$\frac{\partial u}{\partial x}+\frac{\partial v}{\partial y}=0,$$

(2) $$\rho_{f}\left(u\frac{\partial u}{\partial x}+v\frac{\partial u}{\partial y}+2Ωw\right)-\mu \left(\frac{\partial^{2}u}{\partial x^{2}}+\frac{\partial^{2}u}{\partial y^{2}}\right)=-\frac{\partial p}{\partial x}-\sigma B^{2}u,$$

(3) $$\rho_{f}\left(u\frac{\partial v}{\partial x}+v\frac{\partial v}{\partial y}\right)=-\frac{\partial p}{\partial y}+\mu \left(\frac{\partial^{2}v}{\partial x^{2}}+\frac{\partial^{2}v}{\partial y^{2}}\right),$$

(4) $$\rho_{f}\left(u\frac{\partial w}{\partial x}+v\frac{\partial w}{\partial y}-2Ωw\right)=\mu \left(\frac{\partial^{2}w}{\partial x^{2}}+\frac{\partial^{2}w}{\partial y^{2}}\right)-\sigma B^{2}w,$$

(5) $$u\frac{\partial T}{\partial x}+v\frac{\partial T}{\partial y}=\alpha \left(\frac{\partial^{2}T}{\partial x^{2}}+\frac{\partial^{2}T}{\partial y^{2}}\right)+\frac{{\left(\rho c_{P}\right)}_{p}}{{\left(\rho c_{P}\right)}_{f}}\left[\begin{array}{c} D_{B}\left\{\frac{\partial C}{\partial x}\cdot \frac{\partial T}{\partial x}+\frac{\partial C}{\partial y}\cdot \frac{\partial T}{\partial y}\right\}\\ +\left(D_{T}/T_{0}\right)\left\{{\left(\frac{\partial T}{\partial x}\right)}^{2}+{\left(\frac{\partial T}{\partial y}\right)}^{2}\right\} \end{array}\right],$$

(6) $$u\frac{\partial C}{\partial x}+v\frac{\partial C}{\partial y}-\left(\frac{D_{T}}{T_{0}}\right)\left\{\frac{\partial^{2}T}{\partial x^{2}}+\frac{\partial^{2}T}{\partial y^{2}}\right\}=D_{B}\left(\frac{\partial^{2}C}{\partial x^{2}}+\frac{\partial^{2}C}{\partial y^{2}}\right),$$

here, *u* and *v* are the components of velocity along the *x* and *y* axes. *P* is pressure, *T* shows the temperature, *C* is concentration, μ denotes the dynamic viscosity, $\rho_{f}$ is the density of the base fluid, *k* is the thermal conductivity, $D_{B}$ is the coefficient of diffusion, and $c_{p}$ is the specific heat of the nanofluid. The corresponding boundary conditions of the problem are

(7) $$\begin{array}{cc} \; & u=v=w=0,\;T=T_{0},\;C=C_{0}\;\;\mathrm{at}\;y=L,\\ \; & u=ax,v=w=0,\;T=T_{L},\;C=C_{L}\;\;\mathrm{at}\;y=0. \end{array}$$

Introducingthe following dimensionless parameters [44]

(8) $$\zeta =\frac{y}{L},\;u=axf^{\prime }\left(\zeta \right),\;v=-aLf\left(\zeta \right),\;w=axg\left(\zeta \right),\;\theta =\frac{T-T_{L}}{T_{0}-T_{L}},\;\phi =\frac{C-C_{L}}{C_{0}-C_{L}},$$

substituting the above variables in Equations (1)–(6) will result in the system of ordinary differential equations, which are given as [45,46],

(9) $$f^{iv}-R\left(f^{\prime }f^{\prime \prime }-ff^{\prime \prime \prime }\right)-2krg^{\prime }-Mf^{\prime \prime }=0,$$

(10) $$g^{\prime \prime }-R\left(f^{\prime }g-fg^{\prime }\right)+2krf^{\prime }-Mg=0,$$

(11) $$\theta^{\prime \prime }+\mathrm{Pr}\cdot R\cdot f\theta^{\prime }+Nb\phi^{\prime }\theta^{\prime }+Nt\theta^{\prime 2}=0,$$

(12) $$\phi^{\prime \prime }+R.Sc.f\phi^{\prime }+\frac{Nt}{Nb}\theta^{\prime \prime }=0,$$

subjected to boundary conditions

(13) $$\begin{array}{ccc} f\left(0\right)=0,f^{\prime }\left(0\right)=1, & g(0)=0, & \theta (0)=\phi (0)=1,\\ f\left(1\right)=0,f^{\prime }\left(1\right)=0, & g(1)=0, & \theta (1)=\phi (1)=0. \end{array}$$

where, Nt, Nb, Sc, Pr, kr, *M*, and *R*, are the thermophoresis parameter, Brownian motion parameter, Schmidt number, Prandtl number, rotation parameter, magnetic parameter, and viscosity parameter, which are defined as

(14) $$\mathrm{Pr}=\frac{\mu }{\rho_{f}\alpha },\;R=\frac{aL}{v},\;M=\frac{\sigma B^{2}L^{2}}{\rho v},\;kr=\frac{ΩL^{2}}{v},\;Sc=\frac{\mu }{\rho_{f}D},$$

(15) $$Nb={\left(\rho c\right)}_{p}D_{B}\left(C_{L}\right)/\left({\left(\rho c\right)}_{f}\alpha \right),\;Nt={\left(\rho c\right)}_{p}D_{T}\left(T_{L}\right)/\left[{\left(\rho c\right)}_{f}\alpha T_{c}\right].$$

The Nusselt number (Nu) and the specific heat over the bottom wall are defined as

(16) $$Nu=\left|\theta^{\prime }\left(0\right)\right|,C_{f}=\left|f^{\prime \prime }\left(0\right)\right|.$$

## 3. Design Methodology

This section first illustrates the basic structure of a feed-forward neural network and then discusses an optimization technique for the learning procedure of neurons in FFNN architecture.

### 3.1. Feedforward Artificial Neural Networks

In 1943, McCulloch [47] introduced a computational model based on the human brain, which initiated the exploration of artificial neural networks (ANN). ANNs can learn, recognize, and deal with a broad spectrum of complex problems. Feed-forward neural networks (FFNNs) are the only ANN models universally utilized in many feasible applications. An FFNN’s architectural representation makes it appealing because it allows for the identification of a computational model (a function) in structural/network form. Moreover, it is the framework of an FFNN that makes it a prevalent function approximator, which has the effect of approximating and finding solutions to any function or problem [48].

**Figure 2:**
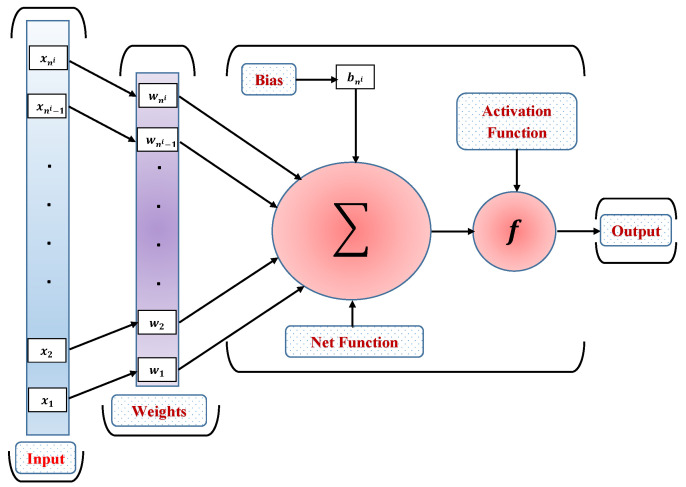
Architecture of a three-layer feed-forward neural network.

An FFNN is a computational model of numerous neurons coupled by weights and stacked layer-by-layer. Thus, FFNNs feature a unique structural architecture in which nodes in one layer have forward connections to nodes in the next layer, as shown in Figure 2. A node of an FFNN can deal with information prevailing on the connection weights. Mathematically, the output $y_{i}$ of a node is computed as:

(17) $$y_{i}=\Theta_{i}\left(\sum_{j=1}^{n^{i}}w_{j}x_{j}+b_{j}\right),$$

where, $x_{i}$ is the input data, $n^{i}$ is the number of sample data, $w_{i}$ are the connection weights, $b^{i}$ is the bias vector and Θ(.) is an activation function. The activation function Θ(x,w) is parameterized with $n^{i}$-dimensional input and weighted vectors as $x_{1},x_{2},x_{3}\cdots ,x_{n^{i}}$ and $w_{1},w_{2},w_{3},\cdots ,w_{n^{i}}$, respectively. Here, the S-shaped curved sigmoid function is used as the activation function, which is given as

(18) $$\Theta \left(x\right)=\frac{1}{1+e^{-(wx+b)}}.$$

The motivation for using a log-sigmoid activation function is that it produces a smooth gradient, which prevents jumping in output values.

### 3.2. Learning Procedure

**Figure 3:**
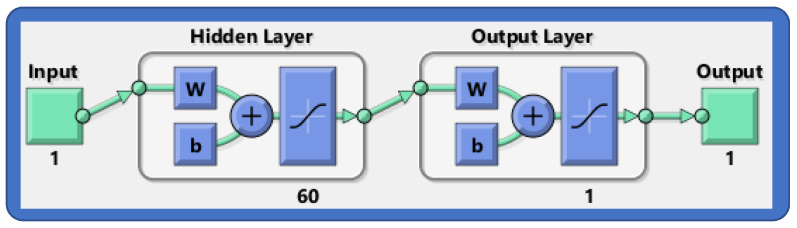
Structure of FFNN model for the modeling of approximate solutions.

**Figure 4:**
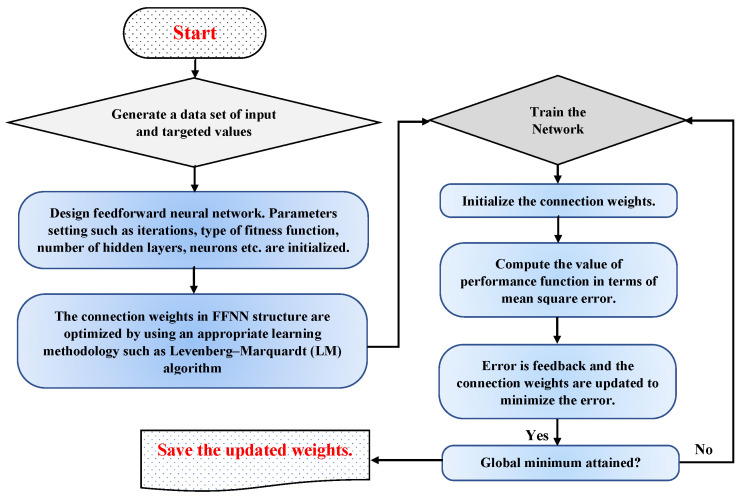
Work flow of the design algorithm.

This section discusses the learning procedure adopted to optimize the connection weights in the FFNN structure for the approximate solutions of the model. Initially, a data set/target data (reference solution) of 1001 points is created using the Adams method in Mathematica. After that, the FNN model is set up with appropriate settings, such as the number of hidden neurons, iteration, and choice of the activation function. The FFNN is then supplied with input (η∈[0,1]) and target data for the supervised machine learning process. The architecture of the FFNN model is shown in Figure 3. In the supervised learning procedure, an objective function is constructed in terms of the mean square that poses a least-squares minimization problem which is given as

(19) $$Minimize\;\mathrm{MSE}=\frac{1}{m}\sum_{j=1}^{m}{\left(y_{j}\left(t\right)-{\hat{y}}_{j}\left(t\right)\right)}^{2},$$

here, $y_{j}\left(t\right)$ is the reference solution and ${\hat{y}}_{j}\left(t\right)$ is the updated/new solution. Furthermore, the optimization technique, such as a back-propagated Levenberg–Marquardt (BLM) algorithm, is utilized to optimize the connection weights by minimizing the performance function given in Equation (19). For perfect modeling of the approximate solutions, the value of MSE approaches zero. The BLM algorithm is a curve-fitting and iterative strategy for locating the minimum of a multivariate function defined as the sum of squares of non-linear real-valued functions. It has become a standard technique for non-linear least-squares problems, with widespread application across a wide range of disciplines. BLM is a mixture of the steepest descent and the Gauss–Newton method. When the current solution is far from optimal, the algorithm behaves like a steepest descent method: sluggish but certain to converge. The Gauss–Newton method is used when the current solution is near to the optimal solution. Some recent applications of the LM algorithm include the solution of an inverse heat conduction problem [49], a system of reaction-diffusion equations in a micro-disk biosensor [50], heat flux estimation [51], energy-gradient fitting [52], charge estimation of lithium-ion batteries [53] and piles embedded in sandy soil [54]. A detailed summary of the working process of a FFNN–BLM algorithm is shown in Figure 4.

## 4. Numerical Experimentation and Discussion

**Figure 5:**
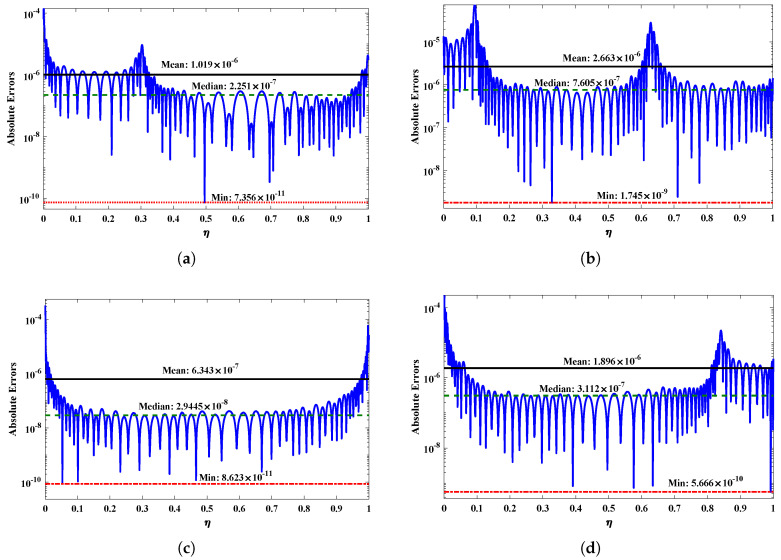
(**a**–**d**) Absolute errors obtained by the FFNN–BLM technique for different profiles of nanofluid. Black, green and red lines correspond to the mean, median and minimum values of absolute errors. (**a**) f(η), (**b**) g(η), (**c**) ϕ(η), (**d**) ϕ(η).

In this section, the proposed FFNN–BLM algorithm is employed to study the influence of variations in the magnetic parameter, Prandtl number, rotation parameter, thermophoresis, and Brownian motion parameter on heat transfer, velocity, gravitational acceleration, and concentration profiles of the nanofluid, governed by Equations (9)–(12). To demonstrate the accuracy and efficiency of the design algorithm, the results obtained by the FFNN–BLM algorithm are compared with the Runge–Kutta–Fehlberg method, the least square method [55], and a machine learning algorithm (NARX-BLM) [56], as detailed in Table 1 and Table 2. The statistics demonstrate the validity of the FFNN–BLM algorithm, and it is observed that the solutions overlap the numerical results with minimal absolute errors that lie around $10^{-5}$ to $10^{-9}$. Figure 5 shows that the average percentage errors for the velocity, acceleration, temperature, and concentration profiles lie around $10^{-5}$ to $10^{-7}$, respectively.

**Figure 6:**
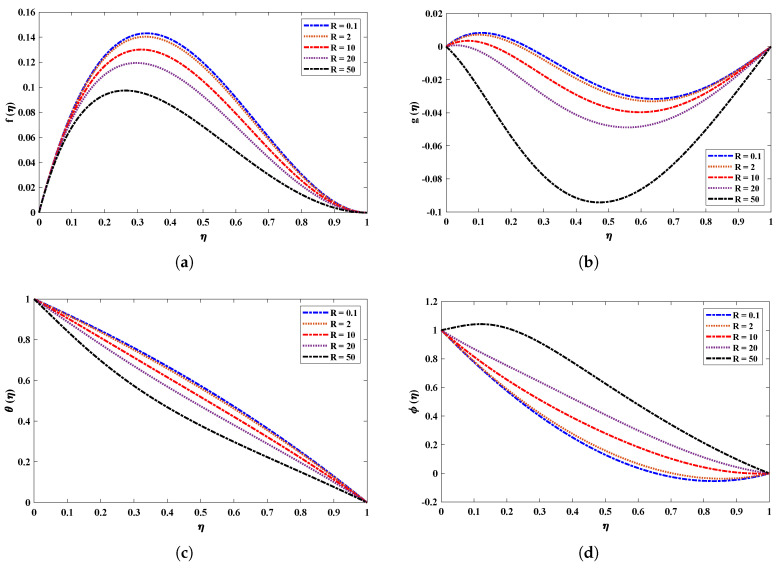
(**a**–**d**) Effect of the viscosity parameter on different profiles of nanofluid when $N_{b}=0.1,\;$$k_{r}\;=\;1,\;M=2,Pr=0.5,\;N_{t}=0.5$ and, Sc=1.

**Figure 7:**
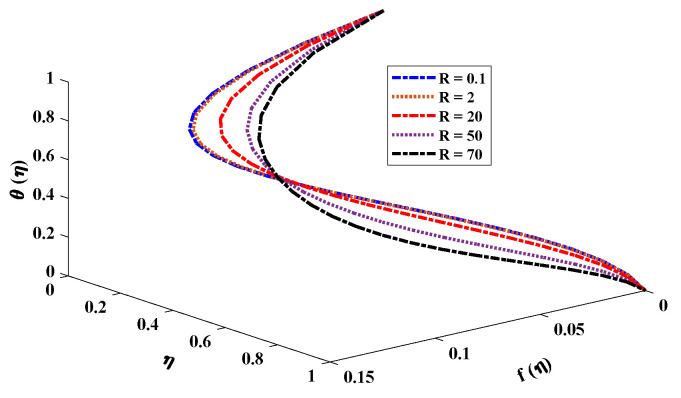
Combined effect of the viscosity parameter on the velocity and temperature profile of the nanofluid with $k_{r}=10,\;M=2,\;N_{b}=0.1,\;Pr=0.5,\;N_{t}=0.5,\;Sc=1$.

**Figure 8:**
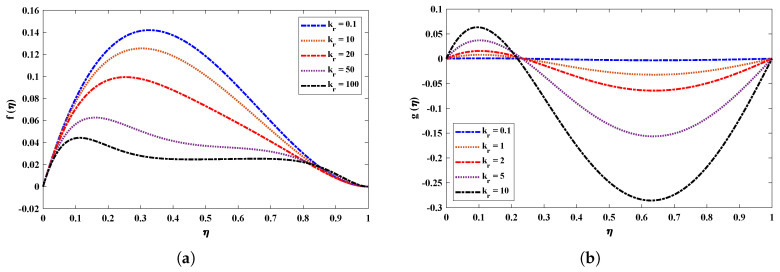
(**a**,**b**) Effect of variations in the rotation parameter on f(η) and g(η) of nanofluid when $N_{b}=0.1,\;R=1,\;Sc=1M=2,\;N_{t}=0.5$ and, Pr=0.5.

Figure 6 and Figure 7 illustrate the viscosity parameter’s effect on various nanofluid profiles. Viscosity is the quantitative measure of the fluid’s resistance to flow. It determines the fluid strain generated by applied shear stress caused by the forces between molecules of the liquid. A fluid’s velocity through porous media is inversely proportional to the viscosity. It is observed, therefore, that an increase in viscosity decreases the horizontal velocity boundary layer thickness of a nanofluid rotating between two horizontal plates. In addition, larger values of *R* imply a higher temperature difference between the surface and the ambient fluid, which decreases the temperature of the fluid. Moreover, the inverse treatment is observed for the concentration of nanoparticles in the fluid. Figure 8 displays the effect of the rotation parameter on the nanofluid; it is evident that if the rotation parameter increases, the fluid’s velocity will decrease gradually and will slightly increase as it follows the boundary.

**Figure 9:**
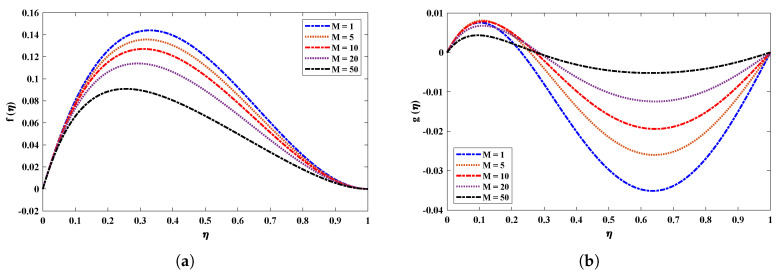
(**a**,**b**) Influence on different profiles of nanofluid by varying magnetic field when R=1, $k_{r}=1,\;Pr=0.5,\;N_{b}=0.1,\;N_{t}=0.1,\;Sc=1$.

**Figure 10:**
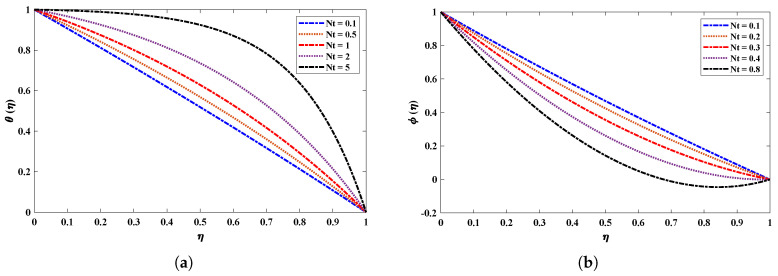
(**a**,**b**) Effect of variation in the thermophoresis parameter on different profiles of nanofluid when $Sc=1,\;k_{r}=1,\;M=2,\;N_{b}=0.1,\;Pr=0.5,\;R=1$.

**Figure 11:**
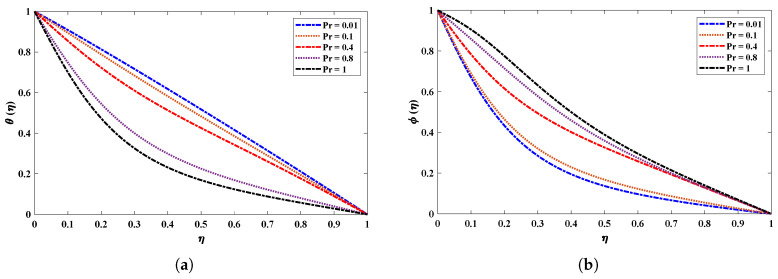
(**a**,**b**) Influence on temperature and concentration profiles of nanofluid by varying the Prandtl number when $R=50,\;k_{r}=1,\;M=2,\;N_{b}=0.1,\;N_{t}=0.1,\;Sc=1$.

**Figure 12:**
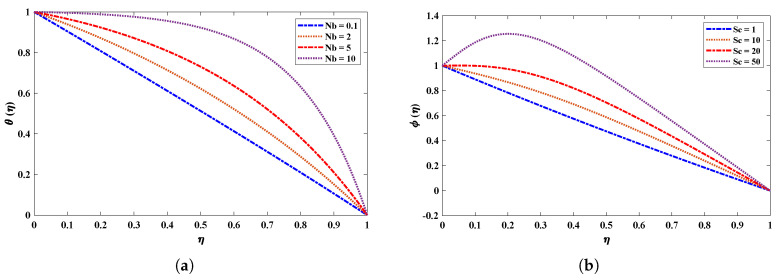
(**a**,**b**) Effects of variations in the Brownian motion parameter and Schmidt number on temperature and concentration profiles of the nanofluid when $Pr=0.5,\;R=1,\;N_{t}=0.1,\;k_{r}=1,\;$$N_{b}=0.1,\;M=2$.

Figure 9 shows the effect of a magnetic field (M) on the velocity and acceleration of the two-phase flow of a nanofluid. The velocity profiles of the fluid deteriorate when the magnetic field parameter increases. This is because increasing the magnetic field causes the opposite force to increase, which is known as the Lorentz force. It is worth noting that the magnetic field’s impact is much stronger on nanofluids than on basic fluids. Figure 10 highlights the effect of the thermophoresis parameter on the temperature and concentration profiles. It is apparent that the escalated values of Nt cause an enrichment in thermophoresis forces which results in the diffusion of nanoparticles which raises the temperature of the nanofluid. In addition, the increase in Nt decreases the concentration of nanoparticles. The influence of the Prandtl number Pr on the temperature distribution is shown in Figure 11. The temperature decreases dramatically as the Prandtl number rises. This corresponds to the physical reality that the thickness of the thermal boundary layer decreases as Pr increases. The most important reason for the increase in nanoparticle concentration is the temperature decrease of the flow field. Figure 12a illustrates the Brownian motion effect on the temperature of the fluid; an increase in Brownian motion leads to a growth in the temperature profile. When the temperature increases (internal energy increases), the particles start moving rapidly. The Schmidt number is a dimensionless number defined as the ratio of momentum diffusivity (viscosity) and mass diffusivity. So, the concentration profile increases with increase in the Schmidt number, as shown in Figure 12b.

**Figure 13:**
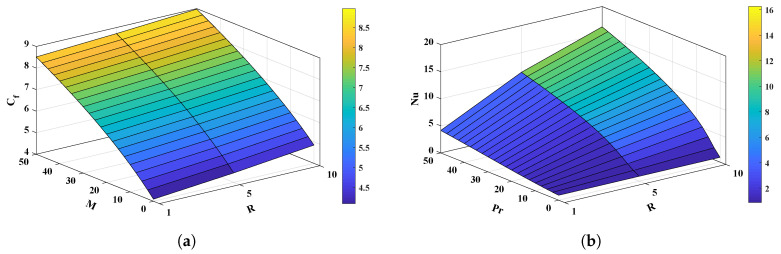
(**a**,**b**) 3-D plots to study the skin-friction coefficient, and Nusselt number (Nu) due to variations in the viscosity parameter, Prandtl number, and magnetic field.

The graphical analysis is conducted as shown in Figure 13 for the influence of variations in various parameters on the Nusselt number (Nu) and the skin-friction coefficient $C_{f}$. The value of the skin-friction coefficient, and of the Nusselt number (Nu) increases with increase in the viscosity parameter, Prandtl number, and the magnetic field.

To check and validate the efficiency, accuracy, and stability of the proposed algorithm, the FFNN–BLM is implemented multiple times to solve Equations (9)–(12) with R=1, Pr=0.5, $Sc=1M=2,\;N_{t}=0.5,\;N_{b}=0.1$, and $k_{r}=10$. Different performance measures are defined to study the convergence and errors in the solutions. The formulation of these indices is given as

(20) $$\left[MSE_{f},MSE_{g}MSE_{\theta },MSE_{\phi }\right]={\left[\begin{array}{c} \frac{1}{M}\sum_{j=1}^{M}{\left(\bar{f}\left(\eta_{j}\right)-f\left(\eta_{j}\right)\right)}^{2},\\ \frac{1}{M}\sum_{j=1}^{M}{\left(\bar{g}\left(\eta_{j}\right)-g\left(\eta_{j}\right)\right)}^{2},\\ \frac{1}{M}\sum_{j=1}^{M}{\left(\bar{\theta }\left(\eta_{j}\right)-\theta \left(\eta_{j}\right)\right)}^{2},\\ \frac{1}{M}\sum_{i=1}^{M}{\left(\bar{\phi }\left(\eta_{j}\right)-\phi \left(\eta_{j}\right)\right)}^{2}, \end{array}\right]}^{t}$$

(21) $$\left[MAD_{f},MAD_{g}MAD_{\theta },MAD_{\phi }\right]={\left[\begin{array}{c} \frac{1}{M}\sum_{j=1}^{M}\left|\bar{f}\left(\eta_{j}\right)-f\left(\eta_{j}\right)\right|,\\ \frac{1}{M}\sum_{j=1}^{M}\left|\bar{g}\left(\eta_{j}\right)-g\left(\eta_{j}\right)\right|,\\ \frac{1}{M}\sum_{j=1}^{M}\left|\bar{\theta }\left(\eta_{j}\right)-\theta \left(\eta_{j}\right)\right|,\\ \frac{1}{M}\sum_{i=1}^{M}\left|\bar{\phi }\left(\eta_{j}\right)-\phi \left(\eta_{j}\right)\right|, \end{array}\right]}^{t}$$

(22) $$\left[{\mathrm{TIC}}_{f},{\mathrm{TIC}}_{g},{\mathrm{TIC}}_{\theta },{\mathrm{TIC}}_{\phi }\right]={\left[\begin{array}{c} \frac{\sqrt{\frac{1}{M}\sum_{j=1}^{M}{\left(\bar{f}\left(\eta_{j}\right)-f\left(\eta_{j}\right)\right)}^{2}}}{\sqrt{\frac{1}{M}\sum_{j=1}^{M}{\left(\bar{f}\left(\eta_{j}\right)\right)}^{2}}+\sqrt{\frac{1}{M}\sum_{j=1}^{M}{\left(f\left(\eta_{j}\right)\right)}^{2}}},\\ \frac{\sqrt{\frac{1}{M}\sum_{j=1}^{M}{\left(\bar{g}\left(\eta_{j}\right)-g\left(\eta_{j}\right)\right)}^{2}}}{\sqrt{\frac{1}{M}\sum_{j=1}^{M}{\left(\bar{g}\left(\eta_{j}\right)\right)}^{2}}+\sqrt{\frac{1}{M}\sum_{j=1}^{M}{\left(g\left(\eta_{j}\right)\right)}^{2}}},\\ \frac{\sqrt{\frac{1}{M}\sum_{j=1}^{M}{\left(\bar{\theta }\left(\eta_{j}\right)-\theta \left(\eta_{j}\right)\right)}^{2}}}{\sqrt{\frac{1}{M}\sum_{j=1}^{M}{\left(\bar{\theta }\left(\eta_{j}\right)\right)}^{2}}+\sqrt{\frac{1}{M}\sum_{j=1}^{M}{\left(\theta \left(\eta_{j}\right)\right)}^{2}}},\\ \frac{\sqrt{\frac{1}{M}\sum_{j=1}^{M}{\left(\bar{\phi }\left(\eta_{j}\right)-\phi \left(\eta_{j}\right)\right)}^{2}}}{\sqrt{\frac{1}{M}\sum_{j=1}^{M}{\left(\bar{\phi }\left(\eta_{j}\right)\right)}^{2}}+\sqrt{\frac{1}{M}\sum_{j=1}^{M}{\left(\phi \left(\eta_{j}\right)\right)}^{2}}}, \end{array}\right]}^{t},$$

(23) $$\left[NSE_{f},NSE_{g},NSE_{\theta },NSE_{\phi }\right]={\left[\begin{array}{cc} 1-\frac{\frac{1}{M}\sum_{j=1}^{M}{\left(\bar{f}\left(\eta_{j}\right)-f\left(\eta_{j}\right)\right)}^{2}}{\sum_{j=1}^{M}{\left(\bar{f}\left(\eta_{j}\right)-\hat{f}\left(\eta_{j}\right)\right)}^{2}}, & \hat{f}\left(\eta_{j}\right)=\frac{1}{M}\sum_{j=1}^{M}f\left(\eta_{j}\right),\\ 1-\frac{\frac{1}{M}\sum_{j=1}^{M}{\left(\bar{g}\left(\eta_{j}\right)-g\left(\eta_{j}\right)\right)}^{2}}{\sum_{j=1}^{M}{\left(\bar{g}\left(\eta_{j}\right)-\hat{g}\left(\eta_{j}\right)\right)}^{2}}, & \hat{g}\left(\eta_{j}\right)=\frac{1}{M}\sum_{j=1}^{M}g\left(\eta_{j}\right),\\ 1-\frac{\frac{1}{M}\sum_{j=1}^{M}{\left(\bar{\theta }\left(\eta_{j}\right)-\theta \left(\eta_{j}\right)\right)}^{2}}{\sum_{j=1}^{M}{\left(\bar{\theta }\left(\eta_{j}\right)-\hat{\theta }\left(\eta_{j}\right)\right)}^{2}}, & \hat{\theta }\left(\eta_{j}\right)=\frac{1}{M}\sum_{j=1}^{M}\theta \left(\eta_{j}\right),\\ 1-\frac{\frac{1}{M}\sum_{j=1}^{M}{\left(\bar{\phi }\left(\eta_{j}\right)-\phi \left(\eta_{j}\right)\right)}^{2}}{\sum_{j=1}^{M}{\left(\bar{\phi }\left(\eta_{j}\right)-\hat{\phi }\left(\eta_{j}\right)\right)}^{2}}, & \hat{\phi }\left(\eta_{j}\right)=\frac{1}{M}\sum_{j=1}^{M}\phi \left(\eta_{j}\right), \end{array}\right]}^{t}$$

(24) $$\left[ENSE_{f},ENSE_{g},ENSE_{\theta },ENSE_{\phi }\right]=\left[1-NSE_{f},1-NSE_{g},1-NSE_{\theta },1-NSE_{\phi }\right].$$

here, $\bar{f}$, $\bar{g}$, $\bar{\theta }$, $\bar{\phi }$ and *f*, *g*, θ, ϕ are the analytical and approximate solutions, respectively. MSE, MAD, TIC and ENSE are the mean square error, mean absolute deviations, Theil’s inequality coefficient (TIC) and the error in Nash–Sutcliffe efficiency (ENSE). The values of these indices should be equal to or approaching zero for perfect modeling.

**Figure 14:**
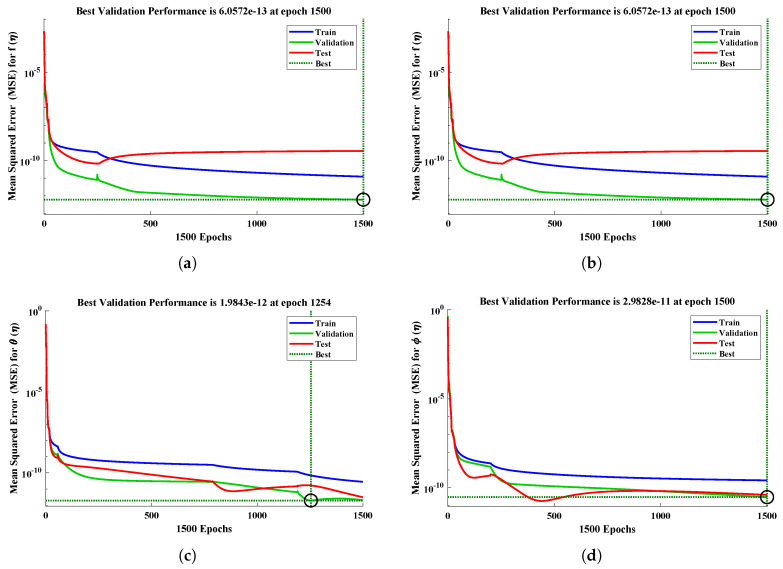
(**a**–**d**) Convergence curves for different profiles of nanofluid with $k_{r}=10,\;$R=1, M=2, $N_{b}=0.1,\;Pr=0.5,\;N_{t}=0.5,\;Sc=1$.

**Figure 15:**
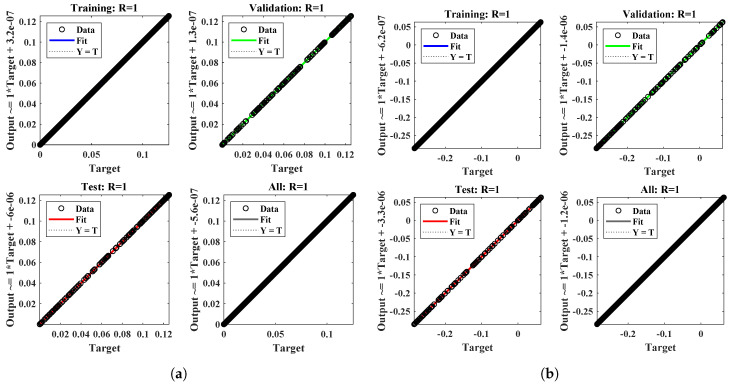

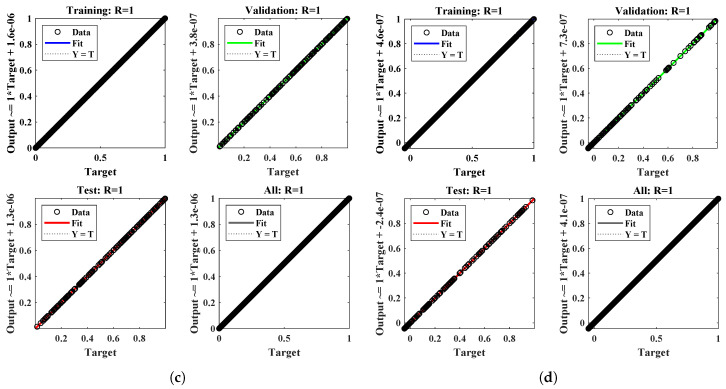
(**a**–**d**) Regression analysis for testing, training and validation data for different profiles of the fluid with $k_{r}=10,\;R=1,\;M=2,N_{b}=0.1,\;Pr=0.5,\;N_{t}=0.5,\;Sc=1$. (**a**) f(η). (**b**) g(η). (**c**) θ(η). (**d**) ϕ(η).

**Figure 16:**
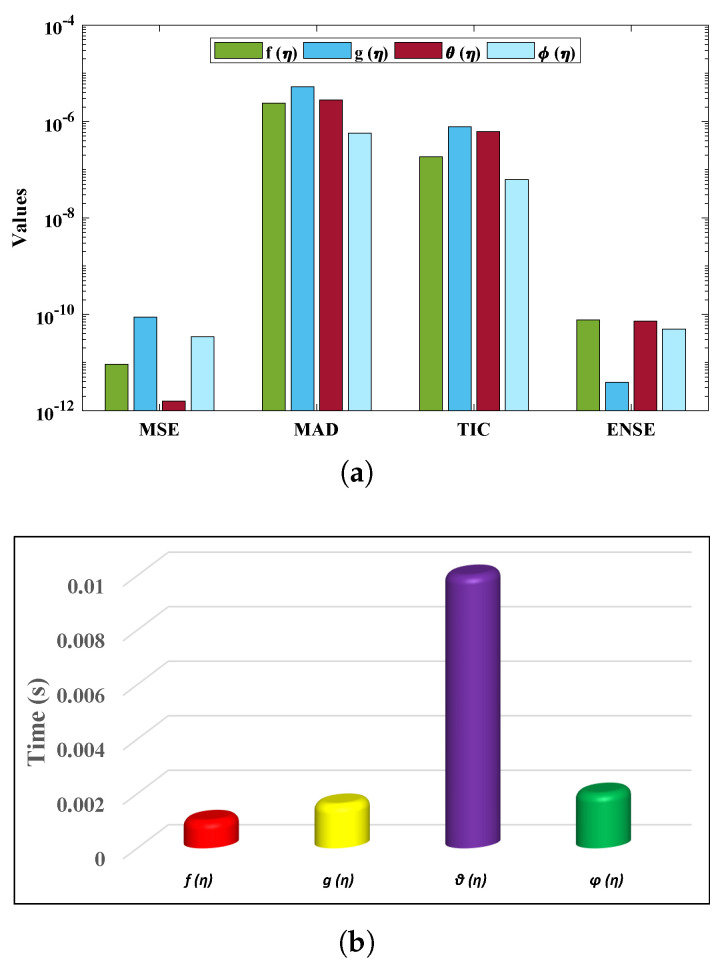
(**a**) Global values of the performance indices, and (**b**) the time taken by the design algorithm in calculating the approximate solutions.

Table 3 shows the minimum and mean values of the performance indicators, along with the standard deviations, when the proposed algorithm is executed for thirty independent runs. It is observed that the minimum values of these indices lie around $10^{-13}$ to $10^{-14}$, $10^{-6}$ to $10^{-8}$, $10^{-7}$ to $10^{-8}$ and $10^{-12}$ to $10^{-13}$, respectively. The standard deviations of the solutions for different profiles of nanofluid are $2.7805\times 10^{-09}$, $1.6772\times 10^{-10}$, $7.0157\times 10^{-10}$ and $4.1393\times 10^{-11}$, respectively, showing the stability of the solutions. The convergence of the objective value in terms of the mean square error for different fluid profiles is shown in Figure 14. The results demonstrate the smoothness of the designed algorithm for solving non-linear problems. The regression models are measured in terms of the correlation coefficients and other related statistical parameters, as shown in Figure 15, to study the strength of fitness of approximate solutions with the reference solutions. The global values of the performance measures for each profile lie between $10^{-6}$ to $10^{-10}$, as demonstrated in Figure 16a. Finally, the computational complexity of the given technique about the minimum time (seconds) taken by the CPU to calculate the solution for each profile is given in Figure 16b. The results demonstrate the robustness of the FFNN–BLM algorithm.

## 5. Conclusions

This study involved an analysis of the two-phase flow of nanofluids between horizontal plates in a rotating system with a magnetic field and external forces. The governing system of non-linear differential equations was solved by utilizing an intelligent strength of artificial neural networks with an optimization algorithm. The velocity f(η), gravitational acceleration (g(η)), temperature (θ(η)) and concentration (ϕ(η)) profiles were investigated under the influence of variations in the magnetic field parameter, Prandtl number, rotation parameter, thermophoresis, and Brownian motion parameter. It was concluded that the increase in viscosity, and rotation parameter, caused an increase in the nanofluid’s velocity profile. Moreover, the fluid’s velocity profile decreased with the magnetic field as it produced the Lorentz force, which opposed the motion and decreased it. In addition, the temperature profile increased with viscosity while a reverse relation was observed with increase in the thermophoresis parameter. It was noted that the velocity boundary layer thickness decreased with increase in the Prandtl number. The concentration profile increased with increase in the Prandtl and Schmidt numbers. Furthermore, the solutions for different profiles of the nanofluid obtained by the FFNN–BLM algorithm were compared with the least square method (LSM), a machine learning algorithm, NARX-LM, and the numerical solutions Runge–Kutta–Fehlberg method (RKFM). The solutions dictated the accuracy of the results by the proposed algorithm in terms of minimum absolute errors. Moreover, to check the validity, stability, and efficiency, the proposed technique was executed for thirty independent runs, and the minimum (Min.), mean and standard deviations (Std. Dev.) of the solutions were calculated for different profiles of the nanofluid in terms of MSE, MAD, TIC, and ENSE. The values of these indices lay around $10^{-8}$ to $10^{-14}$, which demonstrated the perfect modeling of the solutions.

## Figures and Tables

**Table 1:** Validation of the presented results by FFNN–BLM algorithm with LSM and RKF methods for $k_{r}=10,\;R=1,\;M=2,\;N_{b}=0.1,\;Pr=0.5,\;N_{t}=0.5,\;Sc=1$.

	f(η)			g(η)		θ(η)			ϕ(η)	
η	LSM [55]	RKF	FFNN-BLM	RKF	FFNN-BLM	LSM [55]	RKF	FFNN-BLM	RKF	FFNN-BLM
0.00	0	0.00000	0.00000	0.00000	0.00000	1.00000	1.00000	0.99969	1.00000	0.99992
0.05	0.04388	0.04387	0.04387	0.05051	0.05051	0.96205	0.96211	0.96211	0.88623	0.88623
0.10	0.07614	0.07655	0.07655	0.06315	0.06315	0.92299	0.92309	0.92309	0.77832	0.77832
0.15	0.09916	0.09960	0.09960	0.04846	0.04846	0.88280	0.88295	0.88295	0.67642	0.67642
0.20	0.11438	0.11453	0.11453	0.01530	0.01530	0.84148	0.84168	0.84168	0.58066	0.58066
0.25	0.12318	0.12271	0.12271	−0.02900	−0.02900	0.79901	0.79927	0.79927	0.49114	0.49114
0.30	0.12675	0.12540	0.12540	−0.07849	−0.07849	0.75537	0.75569	0.75569	0.40797	0.40797
0.35	0.12607	0.12369	0.12369	−0.12847	−0.12847	0.71054	0.71093	0.71093	0.33126	0.33126
0.40	0.12199	0.11850	0.11850	−0.17525	−0.17525	0.66448	0.66494	0.66494	0.26111	0.26111
0.45	0.11518	0.11064	0.11064	−0.21599	−0.21599	0.61717	0.61769	0.61769	0.19766	0.19766
0.50	0.10622	0.10077	0.10077	−0.24860	−0.24860	0.56856	0.56913	0.56913	0.14105	0.14105
0.55	0.09557	0.08945	0.08945	−0.27153	−0.27153	0.51862	0.51923	0.51923	0.09145	0.09145
0.60	0.08365	0.07718	0.07718	−0.28374	−0.28374	0.46730	0.46793	0.46793	0.04906	0.04906
0.65	0.07084	0.06438	0.06438	−0.28459	−0.28459	0.41454	0.41518	0.41518	0.01410	0.01410
0.70	0.05753	0.05146	0.05146	−0.27380	−0.27380	0.36030	0.36091	0.36091	−0.01321	−0.01321
0.75	0.04417	0.03886	0.03886	−0.25145	−0.25145	0.30451	0.30507	0.30507	−0.03259	−0.03259
0.80	0.03126	0.02704	0.02704	−0.21799	−0.21799	0.24711	0.24759	0.24759	−0.04375	−0.04375
0.85	0.01945	0.01654	0.01654	−0.17434	−0.17434	0.18804	0.18841	0.18841	−0.04640	−0.04640
0.90	0.00957	0.00799	0.00799	−0.12193	−0.12193	0.12721	0.12746	0.12746	−0.04021	−0.04021
0.95	0.00265	0.00217	0.00217	−0.06285	−0.06285	0.06456	0.06468	0.06468	−0.02485	−0.02485
1.00	0.00000	0.00000	0.00000	0.00000	0.00000	0.00000	0.00000	0.00001	0.00000	0.00000

**Table 2:** Comparison of absolute errors in the solutions of the proposed algorithm with LSM and NARX–BLM algorithm for $k_{r}=10,\;R=1,\;M=2,\;N_{b}=0.1,\;Pr=0.5,\;N_{t}=0.5,\;Sc=1$.

	f(η)			g(η)		θ(η)			ϕ(η)	
η	LSM [55]	NARX-BLM	FFNN-BLM	NARX-BLM	FFNN-BLM	LSM [55]	NARX-BLM	FFNN-BLM	NARX-BLM	FFNN-BLM
0.00	0	$1.58\times 10^{-04}$	$1.84\times 10^{-06}$	$3.69\times 10^{-06}$	$6.58\times 10^{-07}$	0	$3.66\times 10^{-04}$	$3.13\times 10^{-04}$	$7.55\times 10^{-04}$	$8.25\times 10^{-05}$
0.05	$1.00\times 10^{-04}$	$6.61\times 10^{-07}$	$3.05\times 10^{-08}$	$2.81\times 10^{-06}$	$2.72\times 10^{-07}$	$5.44\times 10^{-05}$	$3.31\times 10^{-06}$	$6.79\times 10^{-08}$	$1.27\times 10^{-06}$	$4.66\times 10^{-07}$
0.10	$4.00\times 10^{-04}$	$1.38\times 10^{-06}$	$2.33\times 10^{-08}$	$1.11\times 10^{-05}$	$9.27\times 10^{-07}$	$1.00\times 10^{-04}$	$1.12\times 10^{-05}$	$4.79\times 10^{-08}$	$1.64\times 10^{-06}$	$1.82\times 10^{-07}$
0.15	$4.00\times 10^{-04}$	$4.25\times 10^{-07}$	$3.15\times 10^{-08}$	$3.39\times 10^{-07}$	$7.40\times 10^{-09}$	$1.00\times 10^{-04}$	$1.71\times 10^{-06}$	$1.77\times 10^{-08}$	$9.83\times 10^{-07}$	$1.33\times 10^{-07}$
0.20	$1.00\times 10^{-04}$	$1.47\times 10^{-06}$	$2.52\times 10^{-08}$	$2.62\times 10^{-07}$	$9.46\times 10^{-09}$	$2.00\times 10^{-04}$	$1.18\times 10^{-05}$	$5.10\times 10^{-08}$	$4.06\times 10^{-07}$	$2.09\times 10^{-08}$
0.25	$4.00\times 10^{-04}$	$8.64\times 10^{-07}$	$3.94\times 10^{-08}$	$1.43\times 10^{-07}$	$2.26\times 10^{-08}$	$2.00\times 10^{-04}$	$1.18\times 10^{-05}$	$8.46\times 10^{-09}$	$6.73\times 10^{-07}$	$5.66\times 10^{-08}$
0.30	$1.30\times 10^{-03}$	$1.91\times 10^{-06}$	$1.03\times 10^{-06}$	$9.96\times 10^{-07}$	$9.45\times 10^{-08}$	$3.00\times 10^{-04}$	$6.83\times 10^{-06}$	$2.70\times 10^{-08}$	$5.28\times 10^{-07}$	$1.01\times 10^{-07}$
0.35	$2.30\times 10^{-03}$	$1.47\times 10^{-07}$	$4.46\times 10^{-08}$	$3.43\times 10^{-07}$	$1.32\times 10^{-08}$	$3.00\times 10^{-04}$	$4.74\times 10^{-06}$	$2.68\times 10^{-08}$	$1.95\times 10^{-07}$	$8.46\times 10^{-08}$
0.40	$3.40\times 10^{-03}$	$1.27\times 10^{-07}$	$1.77\times 10^{-08}$	$4.77\times 10^{-07}$	$8.16\times 10^{-08}$	$4.00\times 10^{-04}$	$1.10\times 10^{-05}$	$5.65\times 10^{-08}$	$6.62\times 10^{-07}$	$6.19\times 10^{-09}$
0.45	$4.50\times 10^{-03}$	$5.27\times 10^{-07}$	$2.92\times 10^{-08}$	$4.24\times 10^{-07}$	$9.22\times 10^{-08}$	$5.00\times 10^{-04}$	$1.38\times 10^{-06}$	$3.27\times 10^{-09}$	$2.09\times 10^{-07}$	$5.10\times 10^{-08}$
0.50	$5.40\times 10^{-03}$	$5.25\times 10^{-08}$	$2.09\times 10^{-09}$	$5.54\times 10^{-07}$	$7.08\times 10^{-09}$	$5.00\times 10^{-04}$	$7.82\times 10^{-06}$	$8.03\times 10^{-08}$	$1.25\times 10^{-06}$	$8.73\times 10^{-08}$
0.55	$6.10\times 10^{-03}$	$8.53\times 10^{-07}$	$1.49\times 10^{-08}$	$2.67\times 10^{-07}$	$5.42\times 10^{-08}$	$6.00\times 10^{-04}$	$8.30\times 10^{-06}$	$6.51\times 10^{-08}$	$6.88\times 10^{-06}$	$6.01\times 10^{-08}$
0.60	$6.40\times 10^{-03}$	$9.66\times 10^{-07}$	$2.44\times 10^{-09}$	$2.54\times 10^{-07}$	$1.22\times 10^{-07}$	$6.00\times 10^{-04}$	$9.24\times 10^{-06}$	$2.83\times 10^{-08}$	$8.51\times 10^{-07}$	$2.05\times 10^{-07}$
0.65	$6.40\times 10^{-03}$	$6.67\times 10^{-07}$	$4.97\times 10^{-09}$	$8.94\times 10^{-07}$	$7.73\times 10^{-08}$	$6.00\times 10^{-04}$	$7.61\times 10^{-06}$	$4.30\times 10^{-08}$	$5.89\times 10^{-07}$	$1.52\times 10^{-07}$
0.70	$6.00\times 10^{-03}$	$2.32\times 10^{-07}$	$1.56\times 10^{-08}$	$9.65\times 10^{-07}$	$1.35\times 10^{-07}$	$6.00\times 10^{-04}$	$5.79\times 10^{-06}$	$6.11\times 10^{-08}$	$7.83\times 10^{-07}$	$2.95\times 10^{-07}$
0.75	$5.30\times 10^{-03}$	$7.56\times 10^{-08}$	$6.87\times 10^{-09}$	$8.36\times 10^{-07}$	$7.67\times 10^{-08}$	$5.00\times 10^{-04}$	$3.91\times 10^{-07}$	$2.73\times 10^{-08}$	$3.45\times 10^{-07}$	$2.43\times 10^{-08}$
0.80	$4.20\times 10^{-03}$	$4.39\times 10^{-07}$	$2.94\times 10^{-08}$	$5.81\times 10^{-07}$	$2.92\times 10^{-07}$	$4.00\times 10^{-04}$	$1.02\times 10^{-07}$	$6.94\times 10^{-08}$	$2.85\times 10^{-06}$	$1.91\times 10^{-07}$
0.85	$2.90\times 10^{-03}$	$4.28\times 10^{-07}$	$2.56\times 10^{-08}$	$5.97\times 10^{-07}$	$1.53\times 10^{-07}$	$3.00\times 10^{-04}$	$2.87\times 10^{-06}$	$4.37\times 10^{-08}$	$2.78\times 10^{-06}$	$9.78\times 10^{-08}$
0.90	$1.50\times 10^{-03}$	$2.61\times 10^{-07}$	$2.07\times 10^{-08}$	$5.63\times 10^{-06}$	$1.73\times 10^{-07}$	$2.00\times 10^{-04}$	$8.46\times 10^{-06}$	$1.62\times 10^{-08}$	$4.86\times 10^{-07}$	$2.06\times 10^{-07}$
0.95	$4.00\times 10^{-04}$	$8.44\times 10^{-07}$	$2.04\times 10^{-08}$	$8.06\times 10^{-06}$	$3.48\times 10^{-07}$	$1.00\times 10^{-04}$	$3.79\times 10^{-06}$	$2.83\times 10^{-09}$	$7.93\times 10^{-07}$	$2.42\times 10^{-07}$
1.00	0	$1.76\times 10^{-06}$	$6.88\times 10^{-07}$	$1.24\times 10^{-06}$	$2.36\times 10^{-06}$	0	$4.12\times 10^{-04}$	$1.07\times 10^{-05}$	$1.54\times 10^{-06}$	$2.78\times 10^{-07}$

**Table 3:** Statistics of the performance indicators in terms of minimum, mean and standard deviation obtained during the 30 independent executions of the design algorithm for the solutions of f(η), g(η), θ(η) and ϕ(η).

	MSE			MAD			TIC			ENSE		
	Minimum	Mean	Std. Div.	Minimum	Mean	Std. Div.	Minimum	Mean	Std. Div.	Minimum	Mean	Std. Div.
f(η)	$4.7218\times 10^{-14}$	$9.0696\times 10^{-12}$	$5.3115\times 10^{-11}$	$5.2016\times 10^{-07}$	$2.4227\times 10^{-06}$	$3.7123\times 10^{-07}$	$5.0905\times 10^{-08}$	$1.8569\times 10^{-07}$	$1.8004\times 10^{-07}$	$5.0734\times 10^{-13}$	$7.6602\times 10^{-11}$	$2.7805\times 10^{-09}$
g(η)	$8.0125\times 10^{-13}$	$8.7132\times 10^{-11}$	$7.5473\times 10^{-11}$	$3.0559\times 10^{-06}$	$5.2492\times 10^{-06}$	$6.8320\times 10^{-06}$	$7.6598\times 10^{-08}$	$7.7671\times 10^{-07}$	$7.0637\times 10^{-06}$	$5.7247\times 10^{-13}$	$3.8761\times 10^{-12}$	$1.6772\times 10^{-10}$
θ(η)	$2.9421\times 10^{-13}$	$1.5795\times 10^{-12}$	$7.7181\times 10^{-12}$	$1.4253\times 10^{-07}$	$2.7932\times 10^{-06}$	$4.1007\times 10^{-07}$	$5.2329\times 10^{-08}$	$6.1883\times 10^{-07}$	$6.7797\times 10^{-07}$	$7.1346\times 10^{-12}$	$7.1948\times 10^{-11}$	$7.0157\times 10^{-10}$
ϕ(η)	$2.3382\times 10^{-13}$	$3.4396\times 10^{-11}$	$4.4500\times 10^{-10}$	$5.1796\times 10^{-08}$	$5.7441\times 10^{-07}$	$6.1645\times 10^{-07}$	$1.2678\times 10^{-08}$	$6.2865\times 10^{-08}$	$5.9583\times 10^{-08}$	$5.5259\times 10^{-13}$	$4.9361\times 10^{-11}$	$4.1393\times 10^{-11}$

## Data Availability

The data that support the findings of this study are available from the corresponding author upon reasonable request.
